# *Alangium longiflorum* Merr. Leaf Extract Induces Apoptosis in A549 Lung Cancer Cells with Minimal NFκB Transcriptional Activation

**DOI:** 10.31557/APJCP.2020.21.8.2453

**Published:** 2020-08

**Authors:** Cielo Mae D Marquez, Jerremiah G Garcia, Jessica G Antonio, Sonia D Jacinto, Michael C Velarde

**Affiliations:** 1 *Institute of Biology, University of the Philippines Diliman, Quezon City, Philippines. *; 2 *Natural Sciences Research Institute, University of the Philippines Diliman, Quezon City, Philippines. *

**Keywords:** Alangiaceae, cytotoxicity, cardiotoxicity, senescence, zebrafish

## Abstract

The chemotherapy drug doxorubicin (DOX) is effective in treating many types of cancers. However, due to its pro-inflammatory and cardiotoxic side effects, other remedies have also been explored as alternative treatments. The plant *Alangium longiflorum* was reported to contain cytotoxic activity against cancer cells, but it is unclear whether this plant would also yield side effects similar to doxorubicin. Hence, this study investigated cytotoxic activity of *A. longiflorum* leaf extract against lung cancer cells and compared its pro-inflammatory and cardiotoxic side effects with those of DOX.

**Methods:**

Cytotoxic activity of *A. longiflorum* in human lung (A549) and breast (MCF-7) cancer cells was initially assessed by MTT assay and then was compared with doxorubicin. Presence of secondary metabolites in the leaf extract was examined by phytochemical screening. The ability of the plant extract to induce apoptosis was determined by measuring caspase-3/7 activity and apoptosis-related gene expression. Pro-inflammatory response was assessed by quantifying NFκB transcriptional activity and nuclear translocation with dual luciferase reporter and immunofluorescence assays, respectively. Cardiotoxicity was measured using zebrafish as a model organism.

**Results:**

*A. longiflorum *leaf extract displayed high cytotoxic activity against A549 versus MCF-7, which led this study to focus further on A549. Phytochemical screening showed that the extract contained terpenoids, alkaloids, phenols, cardiac glycosides, and tannins. The extract induced apoptosis through activation of caspase-3/7 and upregulation of pro-apoptotic genes without causing NFκB transcriptional activation and nuclear localization. The extract also did not significantly reduce heart function in zebrafish.

**Conclusion:**

Overall, our data suggested that extract from leaves of *A. longiflorum* can have the potential to serve as apoptotic agent towards lung cancer without inducing significant cardiotoxicity.

## Introduction

Chemotherapy drugs, such as doxorubicin (DOX), induce cell death (apoptosis) or permanent growth arrest (cellular senescence) in cancer cells (Gonzalez et al., 2016). Although DOX is effective in preventing cancer progression, it damages normal tissues, including heart tissue, causing structural alterations, left ventricular dysfunction, and congestive heart failure (Cardinale et al., 2015; Maestrini et al., 2017). Hence, cardiotoxicity is a major concern during chemotherapy, as it may lead to morbidity and mortality of cancer patients (Khan et al., 2016). The dosage of DOX may often be reduced to avoid cardiotoxicity (Chang et al., 2017), but it can limit its efficacy. In addition to cardiotoxicity, DOX also induces cellular senescence and activates the inflammatory marker nuclear factor-kappa B (NFκB), creating a pro-inflammatory environment which exacerbates DOX-induced cardiotoxicity (Fallah et al., 2019).

*Alangium longiflorum* (Family: *Alangiaceae*), commonly known as Malatapai, is found in Borneo and the Philippines (Sosef et al., 1998). It is listed as vulnerable on the IUCN Red List (World Conservation Monitoring Centre, 1998). This plant belongs to the genus Alangium, which contains several species used in traditional medicine. While only few studies were conducted on *A. longiflorum*, these studies reported remarkable cytotoxicity of the plant extracts toward cancer cells. For instance, *A. longiflorum* displayed notable cytotoxicity against U251 glioma cells by inhibiting transcriptional activity of hypoxia-induced factor (HIF1), which is activated during hypoxic conditions (Klausmeyer et al., 2008). The alkaloid demethylcephaeline from the stem bark of the plant also exhibited potent cytotoxicity against A549 lung and MCF-7 breast cancer cell lines (Sakurai et al., 2006). 

While *A. longiflorum* extracts have strong cytotoxic activity against cancer cells, it remains unclear whether this natural product would elicit side effects similar to doxorubicin. Hence, this study aimed to determine cytotoxic activity of *A. longiflorum* leaf extract against lung cancer cells and compared its pro-inflammatory and cardiotoxic side effects with those of DOX.

## Materials and Methods

*Plant Material*

*A. longiflorum* leaves (5kg fresh weight) were collected from Mt. Makiling, Los Baños, Laguna on 17 April and 13 October 2017 after obtaining permission from Makiling Center for Mountain Ecosystems and being verified at the Jose Vera Santos Herbarium (Institute of Biology, UP Diliman, voucher specimen no. 21412). Leaves were washed, dried, homogenized, and soaked for 48h in 100mg/ml analytical grade absolute ethanol. Sample was filtered and solvent was removed using a rotary evaporator (Heidolph Hei-Vap) to yield the ethanolic extract (AL). AL was air-dried and oven-dried at 37^o^C prior to use. 

*Phytochemical Screening*

AL was screened for the presence of secondary metabolites as described previously with slight modifications on alkaloid and terpenoid tests (Iqbal et al., 2015; Khanam et al., 2015). For alkaloids, AL was acidified with 1ml 1.5% HCl before adding the Wagner’s reagent. For flavonoids, 1M NaOH was added dropwise to a 1mg/ml of AL in distilled water, followed by addition of 1M HCl to turn the solution colorless if flavonoids are present. Three trials were performed for each phytochemical test.

*Cell Culture Maintenance*

A549 and MCF-7 cells (American Type Culture Collection, Manassas, VA) were maintained in F12 HAMS Nutrient Mixture (Life Technologies 11765-054) and MEM (11095-080), respectively, supplemented with 10% FBS (10500-064) and 1μg/ml gentamicin (Lonza CC-4081J). MCF-7 was also supplemented with 1% insulin-transferrin-selenium A (Thermo Fisher Scientific). Cells were grown in a humidified 5% CO_2_ incubator at 37°C.

*LC*_50_ *and IC*_50_ *Determination*

Cells were seeded overnight at 8,000 to 12,000 cells/well in 96-well plates and exposed to AL (0.02μg/ml to 200μg/ml), DOX (0.000001 – 10μg/ml), and vehicle for 24h or 72h with at least three trials. After incubation, LC_50_ and IC_50_ were evaluated by MTT assay as described previously (Dante et al., 2019). Absorbance was read at 570 nm using Varioskan Flash multimode reader (Thermo Fisher Scientific). 

*Real Time Cell Proliferation Assay*

Cells were seeded overnight at 1,000 cells/well in a 96-well white plate and were treated with vehicle control or LC_50_ values of AL (6.5μg/ml) and DOX (5μg/ml) for 24h done in quadruplicates. Cell proliferation was monitored every 24h for 3 days at 37°C with RealTime-Glo™ MT Cell Viability Assay (Promega) using a Varioskan Flash reader.

*Dual Luciferase Assay*

Cells were seeded overnight in a 6-well plate and transduced with lentiviral NFκB (puro) and CMV-Renilla control (hygro) (Qiagen) reporters at 1TU/cell in antibiotic-free medium for 24h, followed by incubation in 3ml culture medium containing 1μg/ml puromycin and 750μg/ml hygromycin for another 72h. Cells were plated overnight at 8,000 cells/well in 96-well plates and treated with AL and DOX for 72h. Luciferase activities from cell lysates were measured as relative luminescence units (RLU) by Dual-luciferase^® ^assay (Promega) using Varioskan Flash. RLU for firefly luciferase was normalized to RLU of Renilla luciferase. Relative NFκB transcriptional activity was calculated by dividing the normalized RLU for treatment group with their respective vehicle controls. 

*Immunofluorescence*

Cells were seeded overnight at 500 to 2,000 cells/well in 96-well plates and exposed to vehicle and LC_50_ of AL and DOX for 24h, followed by incubation in fresh medium for another 48h. Cells were fixed in 3.7% formaldehyde for 15 minutes, permeabilized with 0.1% Triton X-100 for 10 minutes, and blocked with 1% BSA for 30 minutes at room temperature, with PBS washing in between incubations. Cells were incubated consecutively with NFκB (p65, RelA) antibody (EMD Millipore PC138) (1:500 in 1% BSA) overnight at 4°C, CF™488A secondary antibody (Sigma-Aldrich SAB4600234) (1:1,000 in 1% BSA) for 1h in the dark at room temperature, and 8.12 μM Hoechst 33342 (Thermo Fisher Scientific H3570) for 15 minutes at room temperature, with PBS washing in between incubations. Photomicrographs (n=15) were taken at 200x magnification using an inverted fluorescence microscope (Carl Zeiss™ Axio Vert A.1 with Axiocam IC). 

*Apoptosis Assay*

Cells were treated with LC_50_ of AL or DOX, mitomycin C (MMC, 300 μM) and vehicle for 24h and fixed with 3.7% formaldehyde for 15 minutes at room temperature. Cells were incubated with 7.5μM CellEvent^TM^ Caspase-3/7 substrate reagent for 1h at 37ºC and counterstained with 8.12μM Hoechst 33342 for 15 minutes at room temperature following the manufacturer’s protocol (Invitrogen). Photomicrographs (n=10) were taken at 400x magnification to quantify the percent number of apoptotic cells per treatment.

*RNA Isolation and cDNA Synthesis*

Cells were grown overnight in 6-well plate and treated with AL, DOX, or vehicle for 24h. Total RNA was extracted using TRIzol™ (Invitrogen), quantified by NanoDrop 2000, and assessed for RNA quality under 2% agarose gel electrophoresis. cDNA synthesis was performed using High Capacity cDNA Reverse Transcription Kit (Applied Biosystems), consisting of 2μg RNA, 1X RT buffer, 4mM dNTP mix, 1X random primers, and 50 U Multiscribe^TM^ Reverse Transcriptase in a 20μl reaction mix, as follows: 25ºC for 10 minutes, 37ºC for 120 minutes, and 85ºC for 85 minutes. The resulting amplicons were diluted to a final volume of 100μl nuclease-free water and stored at 20°C. 

*Gene Expression Analysis*

qPCR mastermix contained 1X SYBR Green (Thermo Fisher Scientific), 0.1μM forward and reverse primers (Table 1), and 40ng of cDNA in RNAse/DNAse-free water, as follows: 95°C for 10 minutes, 40 cycles of [95°C for 15 sec, 60°C for 30 sec, 72°C for 30 sec], and melt curve stage of 95°C for 15 sec, 60°C for 1 minute, and 95°C for 15 sec in a StepOne Plus qPCR machine. Relative mRNA expression was calculated using the comparative cycle threshold (CT) values method with beta-actin (ACTB) as housekeeping gene. Three independent trials were performed.

*Zebrafish Cardiotoxicity and Mortality Assays*

Zebrafish experiments were performed at the Institute of Biology, University of the Philippines Diliman, following a protocol approved by the Institutional Animal Care and Use Committee. *Danio rerio* (zebrafish) were obtained from the Freshwater Aquaculture Center, Central Luzon State University, Science City of Muñoz. Spawning was induced by exposing adult fish to light for 1h, following a 12h light-dark cycle. Blastula eggs were incubated for three days at 28ºC in a petri dish containing embryo medium (2.65mM CaCl_2_, 1mM MgSO_4_, 0.8mM NaHCO_3_, and 0.08mM KCl), then treated with AL, DOX, or vehicle for 48h in a 24-well plate at five zebrafish per well in triplicates (n=15). Heart function was assessed by counting heart beats per minute. Percent cumulative mortality was calculated as number of dead fish after two days following treatment, divided by the total number of fish per treatment multiplied by 100. 

*Statistical analysis*

All data were expressed as means **± **SEM. Student’s t-test, one-way, and two-way ANOVA test with post-hoc analysis (Tukey) were done by GraphPad Prism 7. Differences with P-values <0.05 were considered significant. 

**Figure 1:**
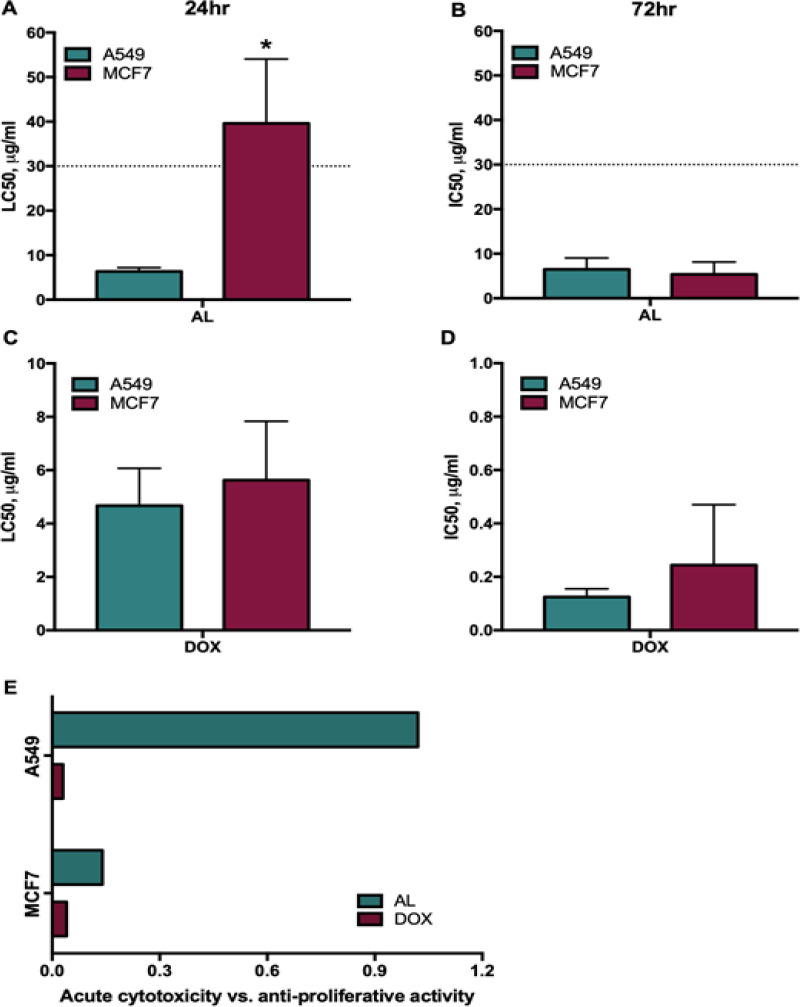
Differential Cytotoxicity and Inhibition of Proliferation in A549 and MCF-7 Cell Lines

**Figure 2:**
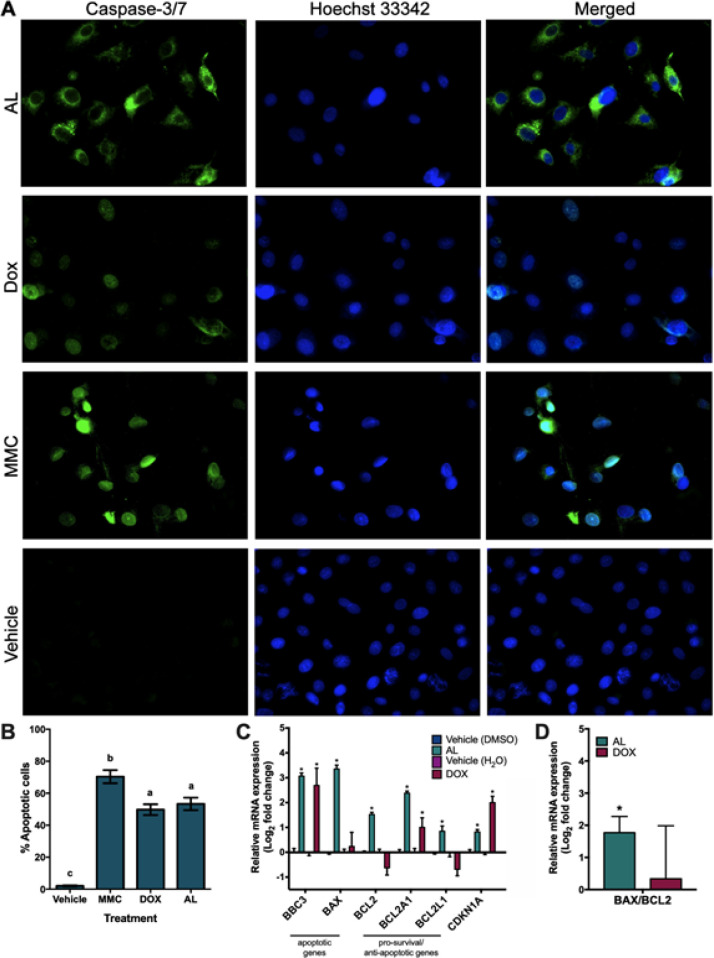
Increased Apoptosis in A549 Cells Treated with AL. (A) Representative fluorescence micrographs of cells. (B) Percent total apoptotic cells. (C) Relative mRNA expression of genes. (D) mRNA expression ratio of *BAX/BCL2. *Different letters and asterisks indicate significant differences at p < 0.05

**Table 1:** Primer Sequence Used for qPCR

Gene Name	Forward Primer (5’to 3’)	Reverse Primer (5’to 3’)
*BBC3­*	TTGTGCTGGTGCCCGTTCCA	AGGCTAGTGGTCACGTTTGGCT
*BAX*	TGATGGACGGGTCCGGG	CACAGGGCCTTGAGCACC
*BCL2*	GGATAACGGAGGCTGGGATG	GACTTCACTTGTGGCCCAG
*BCL2A1*	CAGCAAATTGCCCCGGATG	CCATTTTCCTCTTCTTGTGGG
*BCL2L1*	GAGCTGGTGGTTGACTTTCTC	TCCATCTCCGATTCAGTCCCT
*CDKN1A*	GTTTTCAGGCGCCATGTCAG	CATTAGCGCATCACAGTCGC
*IL6*	CACAAGCGCCTTCGGTCCAG	CATGTCTCCTTTCTCAGGGC
*ACTB*	ACCATGTACCCTGGCATTG	AGGAAAGACACCCACCTTGA

**Figure 3:**
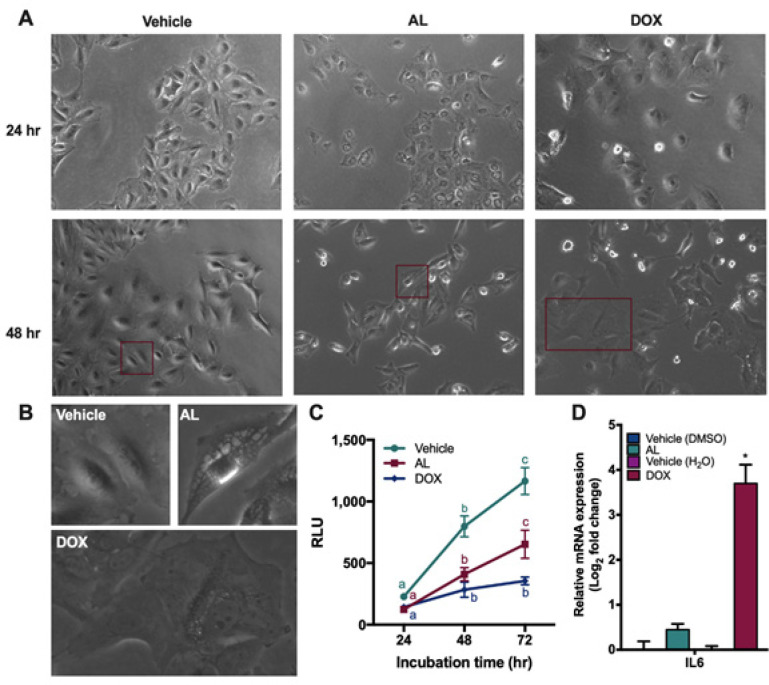
Senescence-Like Phenotype in A549 Cells Treated with DOX but not AL. (A) Photomicrographs of cells at 24h and 48h. (B) Insets of photomicrographs in A. (C) Inhibition of cell proliferation (RLU ± SEM). (D) Relative mRNA expression of IL6. Different letters and asterisks indicate significant differences at *p < 0.05*

**Table 2:** Comparison of LC_50_ and IC_50_ Values of AL and DOX

Treatment	Cancer Cell lines	LC_50_	IC_50_	Acute toxicity vs. anti-proliferative activity
AL	A549	6.34 ± 0.88 μg/ml	6.48 ± 2.57 μg/ml	1.02
	MCF-7	39.61 ± 14.45 μg/ml	5.39 ± 2.74 μg/ml	0.14
DOX	A549	4.67 ± 1.40 μg/ml	0.125 ± 0.03 μg/ml	0.03
	MCF-7	5.62 ± 2.21 μg/ml	0.244 ± 0.226 μg/ml	0.04

LC_50_ and IC_50_ values for each treatment were shown as means **± **SEM. At least three independent trials were performed for each experiment. Ratio of acute toxicity vs. anti-proliferative activity for AL and DOX were included for comparison.

**Figure 4:**
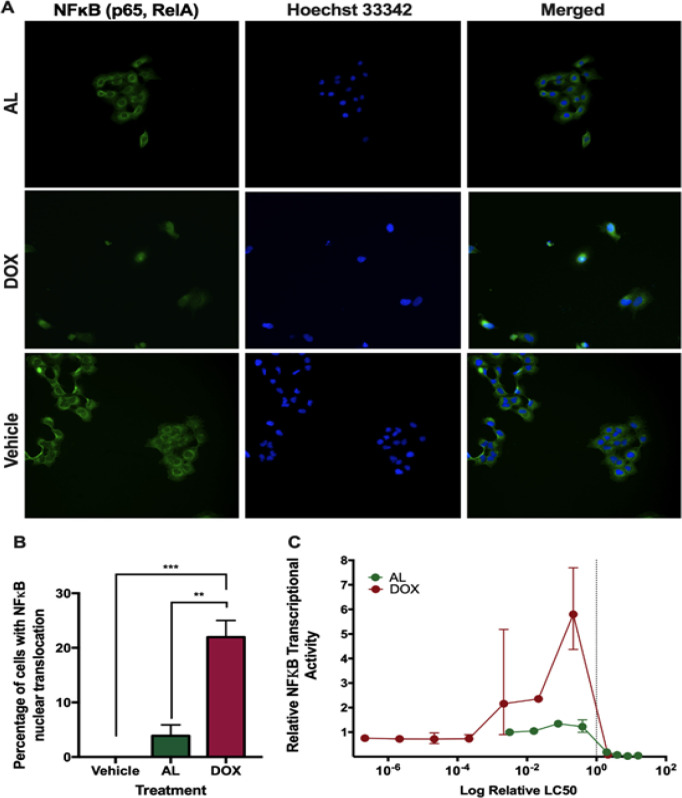
Minimal NFκB Nuclear Translocation and Transcriptional Activity in A549 Treated with AL. (A) Immunofluorescence staining of NFκB. (B) Percentage of cells with nuclear NFκB. (C) Relative NFκB transcriptional activity. Dotted line indicates concentration of treatment relative to its own LC_50_ value. Asterisks indicate significant differences (** p < 0.01) and (*** p < 0.001)

**Table 3:** Phytochemical Screening of Secondary Metabolites in AL

Secondary Metabolites	AL
Saponins	-
Terpenoids	+
Flavonoids	-
Alkaloids (Wagner’s Test)	+
Cardiac glycosides	+
Phenols	+
Tannins	+

Three independent trials were performed for each secondary metabolite tested

**Figure 5:**
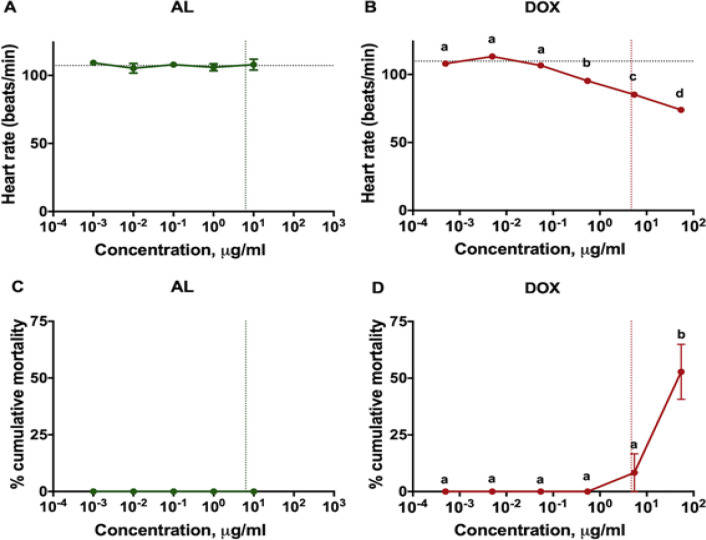
Reduced Heart Function in Zebrafish Treated with DOX. (A,B) Heart rate and (C,D) percent mortality of zebrafish for both treatments compared to vehicle control (horizontal lines). Letters indicate significant differences at p < 0.05. Vertical lines represent LC_50 _of AL and DOX

## Results

*AL induced cytotoxicity*

Lung and breast cancers are the leading types of cancer worldwide (Aggarwal et al., 2016). Hence, this study tested whether AL is effective against A549 lung and MCF-7 breast cancer cells. AL was more cytotoxic against A549 than MCF-7 cells (p = 0.0048), with an LC_50_ of 6.34μg/ml versus 39.61μg/ml, respectively, when treated for 24h (Figure 1A, Table 2). On the other hand, AL inhibited proliferation in A549 and MCF-7 with a similar IC_50_ of 6.48μg/ml and 5.39μg/ml, respectively, when exposed for 72h (p = 0.6558) (Figure 1B, Table 2). Given that the American National Cancer Institute (NCI) has established that crude extracts with LC_50_ or IC_50 _values of ≤30μg/ml are considered cytotoxic and/or anti-proliferative (Abdul-Hafeez et al., 2020), AL may have promising anticancer potential. 

To compare the activity of AL with DOX, the LC_50_ and IC_50_ values of DOX against A549 and MCF-7 were also determined. DOX displayed cytotoxic activity towards A549 and MCF-7 at an LC_50_ of 4.67μg/ml and 5.62μg/ml, respectively, (Figure 1C, Table 2), suggesting that the drug had a similar efficacy with AL in inducing cytotoxicity within 24h (p = 0.3336). However, longer treatment of DOX inhibited proliferation in A549 and MCF-7 at a very low IC_50_ of 125ng/ml and 32.50ng/ml (p = 0.3661), respectively (Figure 1D, Table 2). This suggests that DOX is more potent than AL in inhibiting cancer cell proliferation than inducing cell death. Indeed, AL-treated cells displayed higher acute cytotoxicity versus anti-proliferative index than DOX (Figure 1E), suggesting that the extract induces more A549 cells to undergo cell death than growth arrest. Since A549 was more sensitive to the cytotoxic effect of AL than MCF-7, succeeding bioassays involving AL were focused on A549. Moreover, given that AL induced cytotoxicity in A549 within 24h, LC_50_ at this exposure time was the treatment condition used in succeeding experiments. 

To further assess the classes of secondary metabolites present in the extract, we performed a phytochemical screen. AL extracts contained alkaloids, terpenoids, cardiac glycosides, phenols, and tannins (Table 3), suggesting that these classes of compounds may be involved in cytotoxicity of the plant extract. 

*AL induced apoptosis*

Since apoptosis remains to be the preferred mode of action for cancer therapies (Baig et al., 2016), this study further determined whether AL induces apoptosis in A549.

AL at its LC_50_ increased activation of the apoptosis effector caspase-3/7 in 53.33% of A549 cells within 24h (p < 0.0001) (Figure 2A-B). Likewise, DOX at its LC_50_ caused 49.73% of A459 cells to undergo apoptosis within 24h (p < 0.0001) (Figure 2B). Both treatments were compared with an apoptotic inducer mitomycin C and DMSO to serve as positive and negative controls, respectively. 

Apoptosis is associated with increased expression of the pro-apoptosis gene BBC3 (also called PUMA) (Sonntag et al., 2014) and increased expression ratios of the pro-apoptosis gene BAX over the pro-survival BCL2 gene family (Xu et al., 2016), while inhibition of cell proliferation is associated with increased gene expression of the cell cycle inhibitor cyclin-dependent kinase inhibitor 1A CDKN1A (Karimian et al., 2016). AL significantly increased BBC3 mRNA expression (p < 0.0001) and BAX/BCL2 expression ratios (p = 0.0001) (Figure 2C-D), consistent with its ability to induce apoptosis. In contrast, while DOX significantly increased expression of BBC3 (p = 0.0035) (Figure 2C), it did not significantly increase BAX/BCL2 ratios (p = 0.6302) (Figure 2D). Both AL (p = 0.0002) and DOX (p < 0.0001) increased expression of CDKN1A, suggesting that AL and DOX can inhibit proliferation.

*DOX versus AL for inducing a senescence-like phenotype*

DOX inhibits cancer growth through induction of apoptosis and senescence depending on dose, cell type, and culture conditions (Marino Gammazza et al., 2017; Srdic-Rajic et al., 2017). Indeed, cells treated with DOX showed an enlarged cell phenotype in this study (Figure 3A-B), reminiscent of senescent cells (Neurohr et al., 2019). These cells no longer divided even if DOX was removed from the media (p = 0.0884) (Figure 3C), further confirming that the remaining cells underwent a permanent cell cycle arrest. In contrast, cells treated with AL remained relatively small in size and had several distinctly large vesicles (Figure 3A-B). These remaining cells continued to divide when AL was removed from the media after treatment (p = 0.0027) (Figure 3C). Hence, while AL and DOX both induced apoptosis (Figure 2A-B), DOX but not AL, induced cellular senescence in A549 cells. 

Cellular senescence is a strong tumor suppressor mechanism to limit proliferation of cancer cells. However, senescent cells can also increase expression of pro-inflammatory cytokines, such as IL6 (Coppé et al., 2010; Wiley et al., 2018). Hence, we focused on testing whether AL can also target the same gene. DOX, but not AL, significantly increased IL6 gene expression (p < 0.0001) (Figure 3D), suggesting that AL does not lead to expression of the pro-inflammatory cytokine IL6 (p = 0.0761). Overall, these results suggest that AL can be a strong inducer of apoptosis with minimal IL6 expression, while DOX is an inducer of apoptosis and senescence with IL6 upregulation.

*AL showing minimal NFκB transcriptional activation*

NFκB upregulates expression of pro-inflammatory cytokines, including IL6 (Nan et al., 2018). Hence, this study explored the ability of DOX and AL to activate NFκB. Since activation of NFκB leads to its translocation from the cytoplasm into the nucleus, we checked for subcellular distribution of NFκB by immunofluorescence after treatment with AL and DOX. AL induced NFκB translocation to the nucleus (p = 0.0022), but this was significantly lower than that induced by DOX (p = 0.0008) (Figure 4A-B). 

We further checked for NFκB transcriptional activity using a luciferase reporter assay driven by a promoter containing NFκB response elements. A549 cells treated with AL had lower NFκB transcriptional activity than cells treated with DOX, which yielded the highest activity at a concentration below its LC_50_ value (Figure 4C). Given that NFκB activation is associated with inflammation, the reduced ability of AL versus DOX to induce NFκB nuclear translocation and transcriptional activation suggests that the extract may elicit lower inflammatory side effects than the chemotherapeutic drug. 

*AL and cardiotoxicity*

Heart failure is a serious side effect following DOX treatment (Cardinale et al., 2015; Maestrini et al., 2017), so this study explored the effects of AL on heart function in zebrafish. Heart rate (beats/minute) of zebrafish larvae was used as a parameter for cardiotoxicity. AL extract up to 10μg/ml did not reduce heart rate in zebrafish (p = 0.8467) (Figure 5A). This concentration was above the LC_50_ value of the extract against A549, suggesting that AL induces minimal cardiotoxicity on zebrafish. In contrast, DOX significantly lowered heart rate in zebrafish starting at a concentration of 0.54μg/ml (p = 0.0002) (Figure 5B). This concentration was several folds below the LC_50_ of the drug, confirming the cardiotoxicity of DOX. AL extract also did not cause death in zebrafish larvae across all treatment concentrations (Figure 5C), while DOX induced mortality in some of the fish larvae at ≥LC_50_ concentrations (p = 0.0002) (Figure 5D). The high survival rate of zebrafish within the given range of concentrations suggests minimal overall toxic effect of AL in zebrafish larvae. 

## Discussion

DOX causes topoisomerase-II inhibition, impaired DNA replication, and DNA double strand breaks (Mitry and Edwards, 2016), which can all lead to cellular senescence (Marino Gammazza et al., 2017; Fallah et al., 2019). Indeed, chronic treatment of DOX at low doses can permanently arrest proliferation in MCF-7 cancer cell lines and other cells isolated from different types of solid tumors (Chang et al., 1999; Srdic-Rajic et al., 2017). DOX induces a senescence-like phenotype in cancer cells, resulting in G2/M arrest, increased cell size, and senescence-associated beta-galactosidase activity (Marino Gammazza et al., 2017; Srdic-Rajic et al., 2017). In this study, we showed that AL was more cytotoxic and less likely to induce cellular senescence, as supported by its high efficacy to induce acute cytotoxic versus anti-proliferative activity and inability to increase number of cells with enlarged cell phenotype and gene expression of IL6. 

The ability of DOX to induce senescence may promote inflammation and exacerbate heart dysfunction (Mitry and Edwards, 2016). Indeed, previous studies demonstrated that DOX induced cardiomyopathy and inflammation (Fallah et al., 2019). Consistent with this, we also showed that DOX at several folds below its LC_50_ was sufficient to reduce heart function in zebrafish, as observed in previous findings (Han et al., 2015). This is in stark contrast to AL which did not induce senescence and did not decrease heart function in zebrafish even at concentrations within its LC_50_. While only one parameter was used to assess possible cardiotoxic effect of AL, this study showed that the effective concentration of AL that caused cytotoxicity on cancer cells may only have minimal cardiotoxic side effects in zebrafish.

This study showed that AL was cytotoxic to A549 cells but were more anti-proliferative against MCF-7 cells. The molecular mechanism behind this is still unclear, but it is thought that differences in sensitivity and responsiveness of cancer cells may result from the heterogeneity among cancer cell types and tissue specificity of components of the extract (Tiwary et al., 2015; Baram et al., 2019). MCF-7 contains estrogen receptors which increases cell proliferation and pro-survival signaling upon ligand activation (Jameera Begam et al., 2017). It is interesting to speculate that the presence of phenolic compounds in AL may cause strong growth inhibition in MCF-7, as phenolic compounds can antagonize estrogen receptors (Scott et al., 2016; Pang et al., 2018). Further studies need to be done to investigate the contribution of different components of the extract and to elucidate the mechanism of action between MCF-7 and A549. 

The cytotoxicity of AL in cancer cells was also demonstrated by other studies. For example, the alkaloid 8-hydroxytubulosine from AL leaves displayed broad-spectrum inhibition in a panel of cancer cell lines (Takeuchi et al., 2018). The alkaloid demethylcephaeline from the stem bark of the plant exhibited potent cytotoxicity against A549 and MCF-7 (Sakurai et al., 2006). Tubulosine from the root extract of the same plant also displayed remarkable cytotoxicity against U251 glioma cells by inhibiting transcriptional activity of hypoxia-induced HIF1 (Klausmeyer et al., 2008). While these studies focused on the efficacy of pure alkaloids, it is also worth to consider the possible advantages of using a mixture (i.e. crude extract) over pure isolates. Although numerous single active constituents have been successfully derived from natural products, positive interactions and multi-factorial effects between or among the components present in the crude extract sometimes make the extract more effective than a single compound (Ouhtit et al., 2013; Liu et al., 2016; Kapinova et al., 2017). 

In conclusion, this study showed that AL can be a promising candidate natural product against lung cancer cells by inducing apoptotic signals with minimal expression of pro-inflammatory cytokine. Due to the low cardiotoxicity of AL in zebrafish, further studies should be done to test its potency in eliminating lung cancer in vivo.
